# Oropharyngeal ultrafast ultrasound measurements in mechanically ventilated critically ill patients do not identify post-extubation stridor

**DOI:** 10.1186/s13054-025-05659-2

**Published:** 2025-09-25

**Authors:** Margaux Machefert, Guillaume Prieur, Carlos Díaz López, Claire Dubois, Guillaume Schnell, Elise Artaud-Macari, Bouchra Lamia, Yann Combret, Clément Medrinal

**Affiliations:** 1 Physiotherapy Department, Le Havre Hospital, 76600 Le Havre, France; 2 Erphan UR 20201, Paris-Saclay University, 78000 Versailles, France; 3 Physiotherapy Department, Forcilles Hospital, 77150 Férolles-Attilly, France; 4 EIDUM, Physiotherapy Department, Murcia University, Murcia, Spain; 5 Intensive Care Unit, Le Havre Hospital, 76600 Le Havre, France; 6 GRHVN UR 3830, Rouen Normandie University, 76000 Rouen, France; 7 Intensive Respiratory Care Department, Rouen Hospital, 76000 Rouen, France; 8 Pulmonology Department, Le Havre Hospital, 76600 Le Havre, France

**Keywords:** Post-extubation stridor, Ultrasonography, Shear wave elastography

## Abstract

**Background:**

Post-extubation stridor is a common complication of endotracheal intubation in ICU. This study aimed to assess whether a series of pre-extubation upper airway ultrasound measurements using shear wave elastography (SWE) could help in detecting post-extubation stridor.

**Methods:**

A prospective observational study (NCT05611437) was conducted between 2022 and 2024, which consecutively included 150 adults ICU patients intubated for more than 24 h, without prior surgical or neurological upper airway disease nor swallowing disorders. SWE measurements were performed in the 24 h before extubation. The occurrence of post-extubation stridor, dysphonia and swallowing disorders were assessed within 72 h.

**Results:**

125 participants were included in the final analysis. A total of 2,625 ultrasound images were obtained, with 81% deemed interpretable. Post-extubation stridor occurred in 9% of patients and was independently associated with sepsis at admission (OR 8.98; 95%CI 1.3–62.1). No differences were observed between upper airway ultrasound in patients with or without stridor. Stridor was associated with higher rates of dysphonia (82% vs. 23%), swallowing disorders (36% vs. 11%), and extubation failure (46% vs. 10%). Swallowing disorders were independently associated with the duration of mechanical ventilation (OR 1.10; 95% CI 1.04–1.17). Dysphonia was associated with female sex (OR 3.23; 95%CI 1.24–8.37), sternothyroid muscle stiffness (OR 1.11; 95%CI 1.04–1.18), and days of mechanical ventilation (OR 1.09; 95%CI 1.02–1.15).

**Conclusion:**

Oropharyngeal SWE is feasible in critically ill patients before extubation, but was not predictive of post-extubation stridor. Further studies are needed to explore its role in predicting post-extubation upper airway complications.

**Supplementary Information:**

The online version contains supplementary material available at 10.1186/s13054-025-05659-2.

## Introduction

Post-extubation stridor affects between 4 and 37% of adults ICU patients, and while most cases are mild, stridor is a significant contributor to reintubation in the ICU, with up to 52% of affected patients requiring reintubation within 48 h [1–3]. Laryngeal oedema, typically caused by mucosal injury or prolonged pressure from an endotracheal tube, is the most common mechanism [4]. In ICUs, stridor is associated with longer duration of mechanical ventilation (~ 4 days), increased hospital stays, and higher morbidity and mortality [3, 5, 6].

The cuff leak test (CLT) is the most widely used bedside tool to assess airway patency before extubation [7–9]. Although simple and non-invasive, its predictive performance is inconsistent: specificity is high (~ 90%), but sensitivity varies from 40 to 80%, and thresholds differ between studies, depending on technical and clinical factors [1, 10, 11].

In recent years, upper airway ultrasound has emerged as a promising non-invasive alternative [12]. The air column width difference between deflated and inflated cuff shows potential (sensitivity of 80%, specificity of 81%), but variability and lack of standardisation limit its clinical uptake [12–14]. Ultrafast techniques, such as shear wave elastography (SWE), offer quantitative, operator-independent measurements of tissue stiffness by measuring the shear waves propagation speed [15]. SWE has been successfully applied in critically ill patients and has also demonstrated high reproducibility and sensitivity to subclinical mechanical alterations, including during functional tasks [16, 17]. By detecting stiffness changes from oedema or muscle dysfunction, SWE could provide a novel anatomy-based method for identifying airway compromise prior to extubation. Combining SWE’s established applications in critical care and upper airway assessment remains unexplored.

This study therefore investigated whether SWE measurements of upper airway tissues within 24 h prior to extubation were associated with post-extubation stridor in mechanically ventilated ICU patients. Secondary aims were to explore associations with post-extubation swallowing disorders and dysphonia, given their frequent association, and to describe the practical feasibility and acquisition success of oropharyngeal SWE in the ICU.

## Methods

### Design and setting

This observational study was conducted from September 2022 to December 2024. Ethical approval was obtained from the *Comité de Protection des Personnes* (No. 22.02371.000110), and prospectively registered (NCT05611437). Informed consent was obtained from all participants or their relatives. Full details are provided in supplemental material.

### Participants

We included adults ICU patients ventilated for > 24 h and meeting standard weaning criteria. Non-inclusion criteria included pregnancy, prior laryngeal surgery or radiotherapy, laryngeal tumour, stroke, recurrent nerve palsy, neurological disease, known swallowing disorders, unplanned extubation, tracheostomy, expected death or decision to withhold life sustaining treatments.

### Measurement procedure

**Fig. 1:**
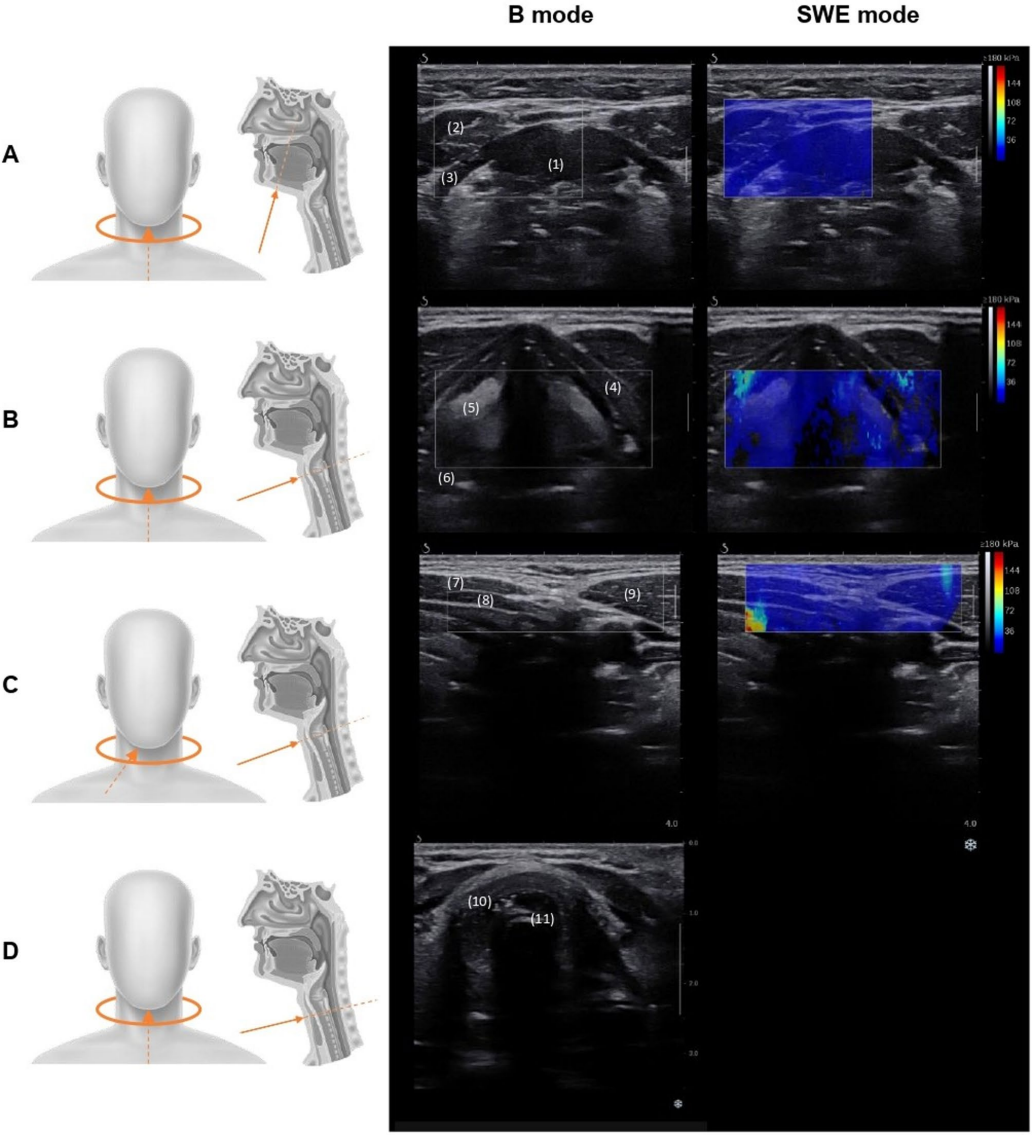
Ultrasound imaging plans showing probe position (left) and images obtained for each plan (right). Images of plans **A**, **B** and **C** were acquired in both **B** and SWE mode, images of plan **D** in **B** mode. Plan **A**: suprahyoid muscles plan – (1) geniohyoid muscle (2), anterior belly of the digastric muscles (3), mylohyoid muscle; Plan **B**: vocal cords plan – (4) thyroid cartilage (5), vocal folds (6), arytenoid cartilage; Plan **C**: infra-hyoid muscles lateral plan – (7) sternohyoid muscle (8), sternothyroid muscle (9), sternocleido-occipito-mastoid muscle; Plan **D**: cricoid cartilage plan – (10) cricoid cartilage (11), intubation tube artefacts

SWE were performed within 24 h before extubation using an Aixplorer scanner (Supersonic Imagine^®^, France) with a 4–15 MHz linear transducer. Participants were examined in a resting semi-recumbent position (30–45° head elevation), neck slightly extended. Sedation protocols were not modified for SWE, but Richmond Agitation-Sedation Scale (RASS) was used to assess agitation and sedation level. Standardised transverse scans were obtained at four anatomical levels: suprahyoid muscles, vocal folds, lateral neck muscles (SCOM, sternothyroid, sternohyoid muscles) and cricoid cartilage (see Fig. 1). We pre-selected ultrasound parameters based on prior evidence, complemented by exploratory measures (see supplemental material). For each site, three images were obtained, and stiffness expressed as Young’s modulus (kPa). Cricoid scans also included measurement of internal diameter of the cricoid cartilage, endotracheal tube position and subglottic secretions [18]. Only images with sufficient quality were retained and the final measurement was calculated as the average of these values. All images were acquired by a single operator (MM) and analysed at the end of the study, by a second operator (CM) who was blinded to patient outcomes and not involved in patient care.

### Characteristics and outcomes

Primary outcome was post-extubation stridor, defined as a high-pitched inspiratory noise within the first 24 h following extubation and requiring medical intervention. Secondary outcomes were swallowing disorders (evaluated trough a rapid assessment considering drooling and mouth closing, deficits in oral/pharyngeal mobility, delayed swallowing, and aspiration) and dysphonia (defined as voice dysfunction in terms of timbre, intensity, hoarseness, or wetness) were also evaluated within 24 h following extubation. Resumption of oral intake at 72 h post-extubation was recorded.

### Sample size calculation

Given limited prior data on ultrasound’s predictive value, the study was powered to detect enough stridor cases for analysis. Based on a literature review estimating a 9% prevalence (*n* = 5,728; 95% CI 7–10%, see Supplemental Methods), we needed 126 participants – anticipating losses: 150 – to capture at least 11 events.

### Statistical analysis

Participants were grouped by presence/absence of post-extubation stridor. Baseline characteristics were compared using chi-squared or Fisher’s exact test for categorical variables, and Student’s t-test or the Mann-Whitney U test for continuous variables. Univariate analyses were performed to identify variables potentially associated with primary (post-extubation stridor) and secondary outcomes (swallowing disorders and dysphonia). Considering the low event rates for certain outcomes, multivariate logistic regressions were constructed parsimoniously, including only variables with clinical relevance and approaching statistical significance in univariate analysis, in accordance with recommendations for event-per-variable ratio in logistic regression. Missing data were handled by multiple imputations (five iterations). Analyses were performed using XLSTAT software, with a two-tailed significance level set at *p* < 0.05.

## Results

### Demographics

**Fig. 2:**
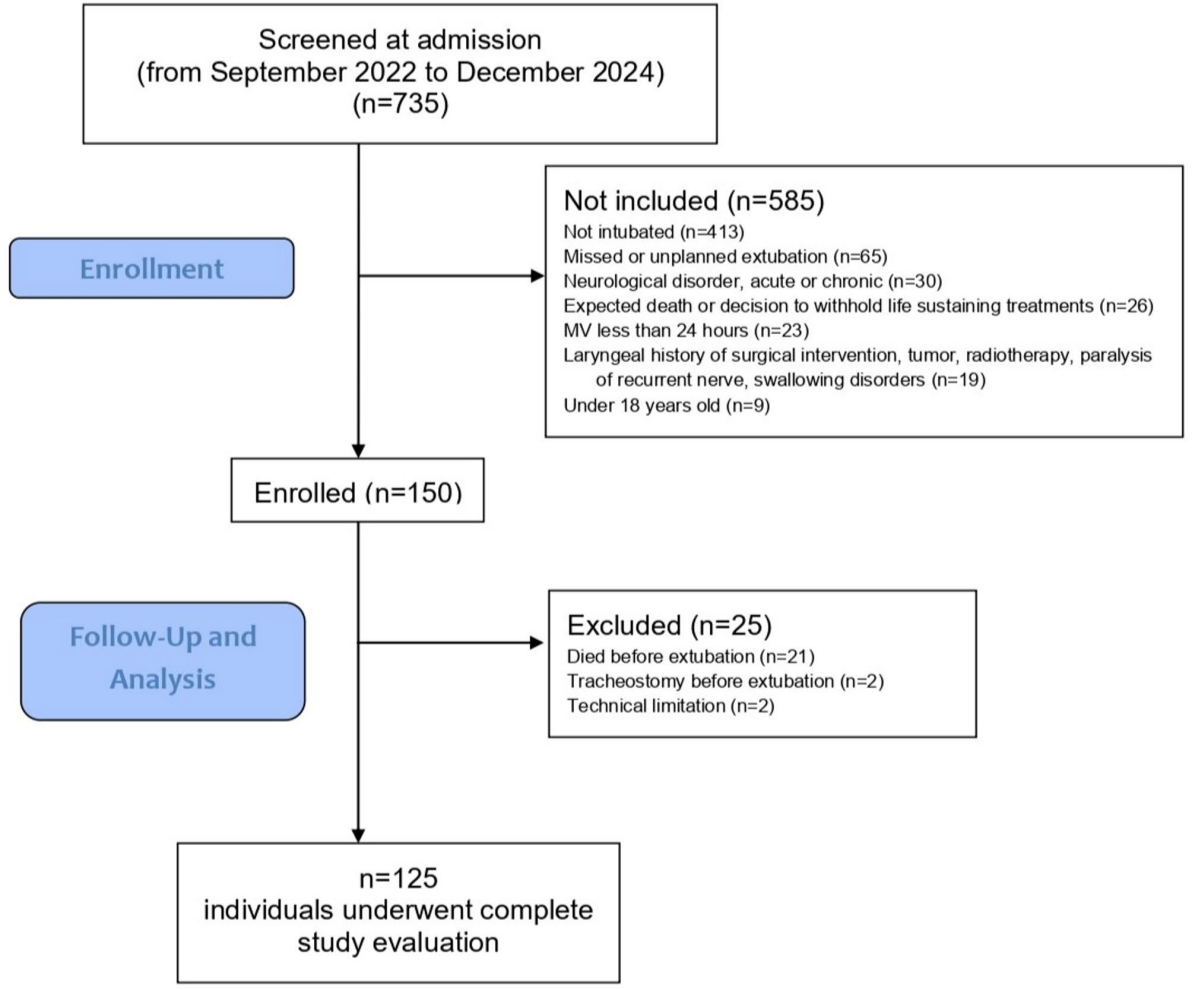
Study flow diagram

Of the 735 ICU admissions between September 2022 and December 2024, 150 participants were enrolled, but only 125 were ultimately included in the final analysis. Figure 2 summarises the study flowchart.

Mean age was 63 ± 19 years, 64% were male, mean BMI was 27.3 ± 8.7 kg/m². The median SAPS II score at admission was 38 ± 23, and the mean duration of mechanical ventilation before inclusion was 8.5 ± 7.5 days (see Table 1). At the time of measurements, most patients were calm and cooperative (RASS = 0; 60%) (details in e-Table 1).

**Table 1:** Cohort characteristics

Variables	Total (*N* = 125)	Post-extubation stridor (*n* = 11)	No post-extubation stridor (*n* = 114)	Mean difference or RR, with 95% CI	*p*-value
**At admission**					
Age, years	63.0 (19.0)	60 (30.5)	64 (17.7)	4 (−7.91 to 15.91)	0.39
Sex, %female	45 (36)	6 (55)	39 (35)	1.59 (0.88 to 2.89)	0.2
Height, cm	170 (13)	165 (27.5)	170 (10)	5 (−2.74 to 12.74)	0.96
Weight, kg	80.0 (26.5)	86.5 (24.5)	79.5 (25.1)	−7 (−22.66 to 8.66)	0.37
BMI, kg/m²	27.3 (8.7)	26.8 (8.05)	27.3 (8.9)	0.5 (−5.02 to 6.02)	0.56
SAPS II, au	38 (23)	44 (29.5)	37.5 (22.5)	−6.5 (−20.97 to 7.97)	0.65
**Main cause of admission**					
Acute cardiac failure	13 (10%)	0 (0%)	13 (11%)	0.38 (0.02 to 6.02)	1
Acute mental status change	18 (14%)	1 (9%)	17 (15%)	0.61 (0.09 to 4.16)	1
Acute neurological disorder	5 (4.0%)	1 (9%)	4 (3%)	2.59 (0.32 to 21.20)	0.33
Acute respiratory failure	46 (37%)	5 (45%)	41 (36%)	1.26 (0.63 to 2.53)	0.53
Drug overdose	5 (4%)	0 (0%)	5 (4%)	0.94 (0.06 to 15.93)	0.46
Monitoring after major surgery	12 (10%)	0 (0%)	12 (10%)	0,41 (0.02 to 6.54)	1
Sepsis	5 (4%)	2 (18%)	3 (3%)	6.9 (1.29 to 37.0)	0.06
Shock	21 (17%)	2 (18%)	19 (17%)	1.09 (0.29 to 4.08)	1
**Co-morbidity**					
Chronic respiratory disease	23 (18%)	0 (0%)	23 (20%)	0.05 (0.01 to 0.32)	0.10
Chronic cardiac insufficiency	13 (10%)	1 (9%)	12 (11%)	0.86 (0.12 to 6.03)	0.88
Hypertension	48 (38%)	5 (46%)	43 (38%)	1.21 (0.61 to 2.40)	0.61
Diabetes	23 (18%)	1 (9%)	22 (19%)	0.47 (0.07 to 3.17)	0.40
Chronic kidney insufficiency	5 (4%)	0 (0%)	5 (4%)	0.21 (0.03 to 1.49)	0.48
Hypercholesterolemia	22 (18%)	1 (9%)	21 (18%)	0.49 (0.07 to 3.33)	0.44
Obesity (BMI ≥ 30)	43 (34%)	4 (36%)	39 (34%)	1.06 (0.47 to 2.42)	0.95
Depression	17 (14%)	3 (27%)	14 (12%)	2.22 (0.75 to 6.56)	0.17
Corticosteroid before extubation	5 (4%)	1 (9%)	4 (4%)	2.59 (0.32 to 21.20)	0.36
**Extubation and follow-up**					
Post-extubation dysphonia	36 (29%)	9 (82%)	27 (23%)	3.45 (2.24 to 5.32)	< 0.001
Post-extubation swallow disorders	34 (27%)	4 (36%)	30 (26%)	1.38 (0.60 to 3.20)	0.35
Dribbling and mouth closure	7 (6%)	1 (9%)	6 (5%)	1.73 (0.23 to 13.08)	0.48
Oral/pharyngeal lack of mobility	16 (13%)	4 (36%)	12 (11%)	3.45 (1.34 to 8.91)	0.03
Latency	21 (17%)	3 (27%)	18 (16%)	1.73 (0.60 to 4.96)	0.39
Aspiration	16 (13%)	0 (0%)	16 (14%)	0.31 (0.02 to 4,49)	0.4
Oral intake resumed at 72 h	69 (55%)	4 (36%)	65 (57%)	0.64 (0.29 to 1.42)	0.18
Extubation failure, re-intubation	15 (12%)	5 (46%)	10 (10%)	5.18 (2.16 to 12.46)	< 0.001
**ICU stay**					
Nb days under MV	8.5 (7.5)	7.5 (4.3)	8.6 (7.7)	1.1 (−3.58 to 5.78)	0.78
Nb days in ICU	16.1 (14.1)	14.2 (8.2)	15.4 (13.9)	1.2 (−7.25 to 9.65)	0.72
Death	13 (11%)	1 (9%)	12 (8%)	0.86 (0.12 to 6.03)	0.37

Data are expressed as mean (SD) and at n (%). Post-extubation stridor, swallowing disorders and dysphonia were assessed within 24 h following mechanical ventilation liberation. au: arbitrary unit; BMI: Body Mass Index; CI: Confidence Interval; ICU: Intensive Care Unit; MV: Mechanical Ventilation; SAPSII: simplified acute physiology score; RR: relative risk

### Ultrasound measurements

A total of 2,625 ultrasound images were collected. Overall, 81% of these were interpretable. The highest interpretability was found in evaluations of the swallowing muscles, secretions, and tube position had, with 91–96% of images deemed usable. In contrast, the lowest scores were for vocal folds and lateral neck muscles, with 71% and 72%, respectively (see Table 2). Non-interpretable images (19%) were primarily due to suboptimal acquisition or incomplete visualisation of the target structures.

**Table 2:** Ultrasonographic images acquisition rate and interpretability

Ultrasonographic images	Total (*N* = 2625)	Suprahyoid muscles (*n* = 750)	Vocal cords (*n* = 375)	Subglottic secretions (*n* = 125)	Tube position (*n* = 250)	Lateral neck muscles (*n* = 1125)
**Usable images**	2134 (81%)	721 (96%)	271 (72%)	120 (96%)	227 (91%)	795 (71%)
**Unusable images**, reason:	491 (19%)	29 (4%)	104 (28%)	5 (4%)	23 (9%)	330 (29%)
- Not achieved	302 (62%)	23 (79%)	39 (38%)	5 (4%)	10 (43%)	225 (68%)
- Blurred	28 (6%)	4 (14%)	7 (7%)	0 (0%)	0 (0%)	17 (5%)
- Well-framed, low echogenicity	61 (12%)	2 (7%)	11 (10%)	0 (0%)	13 (57%)	35 (11%)
- SWE unstable	100 (20%)	0 (0%)	47 (45%)	–	–	53 (16%)

Data are expressed at n (%). Images were undertaken by one trained physiotherapist. SWE: Shearwave Elastography

### Primary outcome – post-extubation stridor

Stridor occurred in 11 out of 125 participants (9%). Univariate analysis showed trends towards association with sepsis at admission (18% vs. 3%; *p* = 0.06) and higher sternothyroid muscle stiffness (12.1 ± 3.6 vs. 10.5 ± 6.7; *p* = 0.16) (see Table 3). However, in the multivariable analysis, only sepsis remained independently associated with post-extubation stridor (OR 8.98; 95%CI 1.3–62.1; *p* = 0.02) (see e-Table 2). Patients with stridor had a higher prevalence of dysphonia (82% vs. 23%; relative risk (RR) 3.45, 95%CI 2.24–5.32; *p* < 0.001), swallowing disorders due to impaired oral or pharyngeal mobility (36% vs. 11%; *p* = 0.03) and extubation failure (46% vs. 10%; RR 5.18, 95%CI 2.16–12.46; *p* < 0.001).

**Table 3:** Sonographic measurements according to stridor status

	Overall (*N* = 125)	Post-extubation stridor (*n* = 11)	No post-extubation stridor (*n* = 114)	*p*-value
**Laryngeal measurements**				
Suprahyoid muscles, (kPa)				
Geniohyoid muscle	9.7 (4.5)	9.2 (4.4)	9.8 (4.5)	0.95
Digastric muscle, anterior belly	8.1 (4.4)	6.5 (5.4)	8.1 (4.4)	0.25
Vocal cords, (kPa)	33.6 (21.9)	33.8 (22.9)	33.6 (21.9)	0.71
Lateral neck muscles, (kPa)				
Sternocleidomastoid muscle	8.8 (5.3)	12.8 (6.1)	8.7 (4.9)	0.35
Sternothyroid muscle	10.6 (6.7)	12.1 (3.6)	10.5 (6.7)	0.16
Sternohyoid muscle	11.4 (8.7)	13.2 (9.2)	11.3 (8.1)	0.22
**Subglottic secretions assessment**				
High	35 (28%)	3 (27%)	32 (28%)	
Moderate	11 (9%)	1 (9%)	10 (9%)	0.99
None	79 (63%)	7 (63%)	72 (63%)	
**Endotracheal tube characteristics**				
Upper airway diameter (mm)	15 (4)	15.4 (3.2)	16.0 (3.8)	0.95
ETT outer diameter (mm)	10.4 (1.1)	10.6 (7.7)	10.4 (0.7)	0.50
Tube-to-airway ratio*	68.9 (15.5)	74.2 (12.6)	68.5 (15.7)	0.19
Tube distance to anterior wall	0.6 (0.3)	0.7 (0.4)	0.6 (0.3)	0.72

Data are expressed as mean (SD) and at n (%). P-values are derived from univariate logistic regression analyses. See e-Table 1 for multivariate results. Subglottic secretions assessment was made on the cricoid plane (see e-Figure1); *Tube-to-airway ratio was calculated as the following ratio: (endotracheal tube outer diameter/cricoid cartilage diameter)*100; CI: Confidence Interval; SD: Standard Deviation; RR: Relative Risk or Risk Ratio; SWE: shear-wave elastography

### Secondary outcomes – dysphagia and dysphonia

Swallowing disorders were present in 27% of participants (see Table 1 for details). Univariate analysis revealed a trend towards association with the duration of mechanical ventilation, sepsis at admission, and patient BMI (see e-Table 3). Multivariable analysis identified the duration of mechanical ventilation as the only independently associated variable (OR 1.10; 95%CI 1.04–1.17; *p* = 0.001) (see e-Table 4).

Post-extubation dysphonia affected 29% of participants. Univariate analysis revealed a trend towards association with female sex and increased sternothyroid muscle stiffness. Trends towards significance were noted for the duration of mechanical ventilation (*p* = 0.07) and the genioglossus muscle stiffness (*p* = 0.07) (see e-Table 5). Multivariable analysis confirmed that the following factors remained independently associated with dysphonia: female sex (OR 3.23; 95%CI 1.24–8.37; *p* = 0.016), increased sternothyroid muscle stiffness (OR 1.11; 95%CI 1.04–1.18; *p* = 0.003), and longer mechanical ventilation (OR 1.09; 95%CI 1.02–1.15; *p* = 0.007) (see e-Table 6).

## Discussion

This prospective observational study was the first to objectively assess the mechanical changes associated with upper airway oedema using a series of pre-extubation SWE measurements, and to determine whether these measurements could help in detecting post-extubation stridor. In our cohort, 9% of participants developed post-extubation stridor, and almost half of these (46%) required reintubation within 72 h. Although none of the ultrasound measurements predicted stridor, several insights emerged: [1] 81% of the 2,625 ultrasound images were interpretable; [2] sepsis at admission was the only independent predictor of stridor; [3] duration of mechanical ventilation was the only independent predictor of swallowing disorders while female sex, sternothyroid muscle stiffness and duration of mechanical ventilation were predictors of dysphonia.

Laryngeal ultrasound is an increasingly explored tool for predicting post-extubation complications, particularly stridor. Although our measurements were inconclusive, the high proportion of interpretable images (81%) indicates that this approach is feasible. The highest interpretability was found for assessments of suprahyoid muscles, supraglottic secretions, and tube-to-airway ratio; lower interpretability rates for the vocal folds and lateral neck muscles. The vocal folds can be obscured in patients with prominent thyroid cartilage and the lateral neck muscles are difficult to delineate and sensitive to probe pressure, presenting specific challenges in these regions [16]. This is the first report to present such a large volume of SWE data in this clinical context. While our study was not designed as a formal feasibility investigation, our findings suggest that the use of oropharyngeal ultrafast ultrasound could be feasible in an ICU setting.

In our cohort, sepsis at admission was the only factor independently associated with post-extubation stridor. Other risk factors commonly described in the literature, such as female sex or duration of mechanical ventilation [2, 4, 19–21] did not reach statistical significance, nor did local factors such as tube-to-airway mismatch [21]. Although sepsis is not a well-established risk factor, the systemic inflammatory response it induces may contribute to, or exacerbate, laryngeal oedema. Moreover, sepsis often leads to haemodynamic or respiratory instability, which delays extubation and increases the duration of mechanical ventilation duration – which is a recognised risk factor for stridor. The lack of significant associations between ultrasound stiffness measurements and stridor could be explained by several technical and physiological factors. Post-extubation stridor can result from diverse upper airway injuries. Brodsky and al. [22] described several grades of laryngeal injury, with more severe grades more likely to cause stridor. Vocal cord ulceration and granulation tissue are found in most, but not all, cases of stridor. Lesions are sometimes located posterior to the vocal folds, at the site of greatest endotracheal tube pressure [4, 23] – a region that is difficult to reach using ultrasound. Stridor was not further investigated in our study due to the absence of systematic endoscopic evaluations. The anatomical challenge, combined with the constraints of our procedure, may have limited our ability to detect such injuries even further. Another key limitation lies in the timing and conditions of SWE measurements. The wide time window in which the measurements were obtained – in the 24 hours prior to extubation – meant they were taken with varying levels of sedation and cooperation, and while the endotracheal tube was still in place and the cuff inflated. This likely limited the relevance of the stiffness values obtained, for two main reasons. Firstly, because of the timing of measurements by itself: any changes in tissue properties occurring closer to or after the endotracheal tube removal, when post-extubation stridor typically manifests, were probably missed; therefore, the absence of SWE changes may reflect a temporal mismatch between measurement and the onset of stridor. Secondly, because the variable participant’s cooperation implied measurements to be taken at rest: this allowed for standardization, but prevented assessment during muscle activation — through phonation or swallowing — when certain abnormalities might be more evident. It is however important to note that the cuff-leak test, the most widely used screening tool for post-extubation stridor, shares these limitations. Typically performed minutes to hours before extubation, it probably also fails to account for delayed-onset oedema. Several studies have shown that patients with a ‘positive’ test may still develop significant stridor afterwards [1, 4, 10]. This highlights a common issue: the difficulty of using a single pre-extubation measurement to predict the development of stridor.

In our study, post-extubation swallowing disorders affected 27% of participants, with mechanical ventilation duration the only independent predictor, consistent with prior studies [24–30]. Post-extubation dysphagia is a clinically relevant ICU complication associated with aspiration, pneumonia, delayed return to oral feeding, and increased length of stay and mortality [31]. Pathophysiological mechanisms are likely multifactorial and remain poorly understood, but prolonged intubation may contribute to the development of dysphagia through mechanisms such as laryngeal desensitisation, disuse atrophy, critical illness-related neuromyopathy or local inflammation [24]. Despite these plausible mechanisms, our study found no significant association between SWE measurements and dysphagia. Although dysphagia is commonly assessed in the ICU [32], clinical or standardized protocols may lack the accuracy required to detect subtle and early impairments. More rigorous diagnostic tools might have classified patients differently, thereby impacting the observed associations, and the real prevalence of swallowing disorders [33, 34]. Additionally, SWE was performed under resting conditions, which may have limited its ability to reveal functional impairments. In swallowing research, hyoid and tongue motion are among the most widely studied parameters and demonstrate strong correlations with dysphagia diagnosis [35–37]; but these dynamics cannot be captured at rest. However, during intubation, tube and cuff mechanically constrain the structures of the aerodigestive tract, preventing normal kinematics and making functional assessment impossible. Current indirect clinical predictors, such as the gag reflex or orofacial motricity, are subjective and inconsistently validated, and more importantly difficult to apply to sedated or uncooperative patients. In contrast, SWE provides quantitative biomechanical information on tissue stiffness that goes beyond what can be achieved with conventional B-mode imaging [38]. Its role in the assessment of functional swallowing remains to be established.

Of the muscles assessed, only increased sternothyroid muscle stiffness was found to be independently associated with post-extubation dysphonia, alongside female sex, and mechanical ventilation duration. These findings are consistent with previous studies that have reported an increased risk of vocal impairment in patients following prolonged intubation [22, 39, 40]. Post-extubation dysphonia has a multifactorial pathophysiology, and the specific role of extrinsic muscle stiffness in voice disorders is unclear. The tone and stiffness of the extrinsic laryngeal muscles influence laryngeal position and tension, thus affecting voice production. Local inflammation, fibrosis, or disuse-related changes could plausibly alter stiffness measurements. Interestingly, no such association was observed with other speech-related muscles, such as the digastric and geniohyoid muscles. This is consistent with the findings of a recent study on primary muscle tension dysphonia, in which SWE failed to detect any significant differences in stiffness in the extrinsic laryngeal muscles of participants with dysphonia compared to healthy controls [16]. The sternothyroid muscle was one of the most technically challenging to assess in our cohort, with a significant proportion of non-interpretable images. While our results suggest a potential link between sternothyroid muscle stiffness and dysphonia, these findings should be interpreted with caution, as further research is needed to clarify the relevance of extrinsic laryngeal muscle properties in post-extubation vocal outcomes.

This study has several limitations that must be acknowledged. Firstly, we did not compare our ultrasound measurements with the cuff leak test (CLT), which is the traditional tool used to predict post-extubation stridor. Future comparative studies should assess SWE alongside established predictors. Despite an appropriate sample size calculation based on expected incidence rates, the relatively low incidence of stridor limited the statistical power of our multivariate analyses, and larger cohort will be required to confirm these findings. Sedation levels were recorded at the time of SWE acquisition using the Richmond Agitation-Sedation Scale (RASS); however, subgroup analysis based on sedation could not be performed due to the small number of patients in the most sedated (RASS − 2) and mildly agitated (RASS + 1) categories. SWE itself remains uncommon in most ICUs, although its adoption is increasing, and it is highly operator-dependent. Even minor variations in probe placement or applied pressure can affect measurement reliability. This underscores the need for standardised acquisition protocols. Additionally, there are currently no reference SWE values available for critically ill patients without upper airway complications. This limits the interpretation of absolute stiffness values in this population. Furthermore, our measurements were obtained within 24 h before extubation, at rest. Incorporating dynamic tasks if feasible, such as phonation or swallowing, or serial assessments at predefined pre- and post-extubation time points would provide a more accurate picture of relevant physiological changes. Addressing these challenges will be essential in determining whether SWE can complement existing screening tools.

Despite these limitations, our study has several strengths. It is the first to report such a large dataset of SWE measurements in the ICU context, comprising over 2,600 images from 125 patients. It demonstrates high image interpretability in this population, and provides the first quantitative SWE dataset in this setting, along with an applicable acquisition protocol for critically ill patients.

In conclusion, SWE of oropharyngeal muscles and vocal folds at multiple anatomical levels before extubation was feasible in critically ill patients. Although the technique failed to predict stridor in our cohort, it did identify clinical associations. These results provide a strong basis for future studies integrating dynamic, serial and multimodal assessments in order to fully evaluate the potential of SWE for the early detection of post-extubation upper airway complications.

## Supplementary Information

Supplementary Material 1

## Data Availability

The datasets used and/or analysed during the current study are available from the corresponding author on reasonable request: Margaux Machefert, [margaux.machefert@ch-havre.fr](mailto: margaux.machefert@ch-havre.fr).

## Abbreviations

CLT: Cuff leak test
CI: Confidence interval
ICU: Intensive care unit
MV: Mechanical ventilation
OR: Odds ratio
RR: Relative risk
SWE: Shear wave elastography
