# Supplementary material for: Oropharyngeal ultrafast ultrasound measurements in mechanically ventilated critically ill patients do not identify post-extubation stridor
Source: Crit Care. 2025 Sep 25;29:406. doi: 10.1186/s13054-025-05659-2 (PMC12465404; doi:10.1186/s13054-025-05659-2)
Supplement: Supplementary file 1 — Supplementary Material 1 [file 13054_2025_5659_MOESM1_ESM.docx]

Supplemental data

[**Methods 2**](#_Toc199755409)

[Design and setting 2](#_Toc199755410)

[Participants 2](#_Toc199755411)

[Measurement Procedure 2](#_Toc199755412)

[**Supplemental Tables 5**](#_Toc199755414)

[**Supplemental Figures 8**](#_Toc199755415)

[**Appendix 1 9**](#_Toc199755416)

# Methods

## **Design and setting**

This observational study was conducted from September 2022 to December 2024. It was approved by the French Comité de Protection des Personnes (Approval N°22.02371.000110), and prospectively registered (NCT05611437). Informed consent was obtained from all participants or their relatives.

## **Participants**

We included adults in ICU, on MV for >24h and filing mechanical ventilation weaning criteria. Exclusion criteria were pregnancy, history of laryngeal tumor, stroke, paralysis of recurrent nerve or swallowing disorders, history of surgical laryngeal intervention or radiotherapy, unplanned extubation, tracheostomy and under guardianship.

## **Measurement Procedure**

Ultrasound imaging and SWE measurements were obtained within 24h prior to the planned extubation. An Aixplorer ultrasound scanner (Supersonic Imagine®, Aix-en-Provence, France) was used with a 4-15Hz linear transducer and musculoskeletal presets. Each test was carried out in accordance with current hygiene regulations (i.e. cleaning of the anatomical area for measurement and probe disinfection after each utilization).

Participants were assessed in supine position, neck extended. After applying a large amount of ultrasound conductive gel for optimal acoustic coupling, the ultrasonographic transducer was placed transversely, with minimal pressure to not distort muscle structure, over three different scanning planes. The rotation and angle of the transducer were then finely adjusted.

Images of the submental muscles were first acquired. Transducer was placed in a coronal place, midway between the anterior of the mandible and superior edge of the hyoid bone, perpendicularly. To ensure correct positioning, the following elements had to be included in the images: the anterior bellies of the two digastric muscles (reminiscent of Mickey's ears), the geniohyoid muscle in the middle (Mickey's head) and the mylohyoid muscle (the band between the ears and the head). Stiffness of the geniohyoid muscle and anterior bellies of the digastric muscles were captured. By activating the SWE mode, a coloured box describing tissue stiffness appeared on the screen over the area of interest. Images were frozen when stable elastography was obtained. On the frozen image, stiffness was measured using the largest area possible on the homogeneous zone of interest. A single Q-Box with a single value reported was performed for each measurement on a stable and frozen image. Young’s modulus was extracted in kPa to describe muscle stiffness.

The probe was then placed transversely on the thyroid cartilage and moved caudo-cranially to visualise the vocal folds in the right axis. To ensure correct positioning, the following elements had to be included in the images: thyroid cartilage (V-shaped appearance), vocal cords and folds, arytenoid cartilages and, if possible, artefacts from the intubation probe. Stiffness of the vocal folds was then captured, following the same protocol.

By moving sideways, holding the probe on the thyroid cartilage, transversely, we decided to capture the stiffness of the lateral muscles of the neck: SCOM, sternothyroid muscle and sternohyoid muscle, lateral to the trachea. We decided to include these muscles, easy to visualise on the surface, and involved in stabilising the hyoid bone for swallowing (i.e. the sternothyroid and sternohyoid muscles), or acting as an inspiratory accessory muscle (i.e. SCOM). Stiffness of the three muscles together was captured, following the same protocol.

The final scan plane was obtained by placing the probe over the cricoid cartilage. To ensure correct positioning, the following elements had to be included in the images: the cricoid cartilage (C-shaped appearance), the thyroid gland and, if possible, the intubation probe. The images were acquired and saved in B mode. We measured the internal diameter of the cartilage and measured the position of the probe in relation to the anterior wall to calculate a ratio of the diameter of the intubation probe to the size of the cricoid cartilage. The presence of subglottic secretions (see e-Figure1) was also assessed at this level (none, moderate, high).

All images were obtained by a single investigator, trained in ultrasound. Images were analysed by two examiners, at the end of the study. For all participants and each scan plane of interest, three different frozen images were taker, to assess reproducibility (12 images/participant, 1800 images). The whole procedure for a measurement session (preparation and all three measurements for different scanning planes) took approximately 10 minutes at the participant’s bedside.

In cases where extubation was delayed beyond 24 hours following imaging, a new set of images was acquired, and only the most recent set within the 24-hour window before actual extubation was included in the final analysis. Image quality was assessed prior to analysis: only images showing clear anatomical structures and stable elastography frames (in SWE mode) were retained. For each anatomical plane, the average of the retained measurements (maximum three per plane) was used for statistical analysis.

## **Parameters selection**

The muscles and anatomical structures selected for ultrasound assessment were chosen based on their functional relevance to the pathophysiology of post-extubation stridor, as well as on their accessibility and the feasibility of imaging them in an ICU setting.

The vocal folds were included as they can be directly affected by the endotracheal tube and cuff, which can lead to localised edema, ulceration, or other injuries that contribute directly to the development of post-extubation stridor.

The infrahyoid group, specifically the sternothyroid and sternohyoid muscles (as the thyrohyoid and omohyoid muscles are small and challenging to image), was included due to their essential roles in stabilising and positioning the larynx and hyoid bone during respiration, phonation, and swallowing. Additionally, the anterior belly of the digastric muscle and the geniohyoid muscle were assessed due to their key roles in elevating the hyoid bone and supporting the floor of the mouth. Together, these muscles contribute to the positioning and dynamic movement of the larynx, and may be affected by oedema, inflammation, or tissue injury resulting from intubation. Changes in their biomechanical properties could serve as indirect markers of upper airway compromise. Our muscle selection also included exploratory parameters, such as the sternocleidomastoid, for which there is limited literature on its role in post-extubation complications.

Parameters such as the tube-to-airway ratio and the presence of supraglottic secretions were incorporated to provide additional information on airway patency and oedema. While the tube-to-airway ratio is primarily studied in paediatric populations, some adult studies have also emphasised the importance of correctly sized endotracheal tubes. As tube size relative to airway dimensions can influence mucosal pressure and subsequent oedema formation, assessing this ratio alongside supraglottic secretions provides valuable insight into the risk of stridor and overall airway function.

Beyond stridor, these anatomical structures were also considered relevant for evaluating secondary outcomes such as dysphagia and dysphonia. The extrinsic laryngeal muscles play an important role in swallowing mechanics and voice production; therefore, alterations in their stiffness or function, as measured by shear wave elastography, may indicate early or subclinical impairments. Similarly, changes in vocal fold biomechanics or supraglottic secretions could affect phonation and airway protection during swallowing, linking these parameters to dysphonia and dysphagia. Thus, assessing these muscles and anatomical features together provides a comprehensive approach to understanding upper airway function and its complications following extubation.

The selected ultrasound parameters therefore reflect an approach aimed at capturing biomechanical and structural changes relevant not only to the primary outcome of post-extubation stridor, but also to secondary complications such as swallowing and voice disorders. This maximises the clinical applicability of this imaging protocol in critically ill patients.

## **Sample size calculation**

A review of 35 studies involving 5,728 patients was conducted to estimate the prevalence of post-extubation stridor, which was found to be 9% (95% CI 7–10%) (see Appendix 1). Using this estimate, we calculated that a sample size of at least 11 stridor events was required to ensure sufficient data for meaningful exploratory analyses. However, due to the exploratory nature of our study and the limited prior evidence on the predictive performance of shear wave elastography (SWE) in this context, the sample size was not designed to evaluate the clinical impact or effectiveness of ultrasound-based screening in reducing adverse outcomes. Instead, the aim was to provide preliminary insights into the feasibility of using SWE and its potential association with post-extubation complications.

# Supplemental Tables

**e-Table1.** Multivariate Logistic Regression Analysis for Stridor Prediction

**e-Table2.** Univariate Poisson Regression Analysis for Swallowing Disorders

**e-Table3.** Multivariate Logistic Regression Analysis for Swallowing Disorders

**e-Table4.** Univariate Poisson Regression Analysis for Dysphonia

**e-Table5.** Multivariate Logistic Regression Analysis for Dysphonia

| Nb of patients | RASS scores at the time of measurements |
| --- | --- |
| 3 (2,4%) | -2 |
| 38 (30,4%) | -1 |
| 75 (60%) | 0 |
| 9 (7,2%) | 1 |
| 125 |  |

e-Table 1. Patients Richmond Agitation-Sedation Scale (RASS) scores during images acquisition.

| Variable | Coefficient (β) | Std. Error | p-value | Odds Ratio | 95% CI (Odds Ratio) |
| --- | --- | --- | --- | --- | --- |
| constant | -2.960 | 0.633 | 0.000 | 0.05 | [0.01; 0.18] |
| Sternothyroid muscle stiffness (kPa) | 0.034 | 0.037 | 0.366 | 1.03 | [0.96; 1.11] |
| Sepsis at admission (yes) | 2.195 | 0.987 | 0.026 | 8.98 | [1.30; 62.10] |

e-Table 2. Multivariate Logistic Regression Analysis for Stridor Prediction

| Variable | RR | 95% CI | p-value |
| --- | --- | --- | --- |
| Age | 0.99 | [0.96–1.02] | 0.98 |
| BMI | 1.77 | [0.97–3.24] | 0.054 |
| SAPS II | 1.01 | [0.98–1.03] | 0.70 |
| Days under mechanical ventilation | 1.09 | [1.03–1.15] | 0.002 |
| Geniohyoid muscle stiffness (kPa) | 1.03 | [0.95–1.13] | 0.46 |
| Digastric muscle stiffness (kPa) | 1.06 | [0.96–1.15] | 0.24 |
| Vocal cords stiffness (kPa) | 0.99 | [0.96–1.01] | 0.24 |
| Cricoid diameter (cm) | 0.83 | [0.25–2.81] | 0.77 |
| Anterior distance (cm) | 3.07 | [0.09–106.78] | 0.54 |
| Tube-to-airway ratio | 0.99 | [0.96–1.01] | 0.40 |
| SCOM muscle stiffness (kPa) | 1.00 | [0.92–1.08] | 0.93 |
| Sternothyroid muscle stiffness (kPa) | 1.05 | [1.01–1.10] | 0.19 |
| Sternohyoid muscle stiffness (kPa) | 0.99 | [0.95–1.05] | 0.98 |
| Sepsis at admission | 6.13 | [1.09–34.6] | 0.04 |

e-Table 3. Univariate Poisson Regression Analysis for Swallowing Disorders.

| Variable | OR | 95% CI | p-value |
| --- | --- | --- | --- |
| BMI | 1.03 | [0.96–1.10] | 0.44 |
| Days under mechanical ventilation | 1.10 | [1.04–1.17] | 0.001 |
| Sternothyroid muscle stiffness (kPa) | 1.04 | [0.99–1.10] | 0.14 |
| Sepsis at admission (yes) | 9.3 | [0.77–112.6] | 0.08 |

e-Table 4. Multivariate Logistic Regression Analysis for Swallowing Disorders.

| Variable | RR | 95% CI | p-value |
| --- | --- | --- | --- |
| Age | 0.98 | [0.95–1.01] | 0.20 |
| BMI | 1.41 | [0.79–2.50] | 0.24 |
| SAPS II | 1.01 | [0.99–1.04] | 0.40 |
| Days under mechanical ventilation | 1.05 | [1.00–1.11] | 0.07 |
| Geniohyoid muscle stiffness (kPa) | 1.06 | [1.00–1.12] | 0.07 |
| Digastric muscle stiffness (kPa) | 0.94 | [0.86–1.03] | 0.15 |
| Vocal cords stiffness (kPa) | 1.00 | [0.98–1.02] | 0.97 |
| Cricoid diameter (cm) | 0.96 | [0.31–2.99] | 0.94 |
| Anterior distance (cm) | 0.86 | [0.02–3.89] | 0.94 |
| Tube-to-airway ratio | 1.00 | [0.98–1.03] | 0.73 |
| SCOM muscle stiffness (kPa) | 1.00 | [0.93–1.07] | 0.92 |
| Sternothyroid muscle stiffness (kPa) | 1.05 | [1.00–1.10] | 0.044 |
| Sternohyoid muscle stiffness (kPa) | 0.99 | [0.95–1.03] | 0.52 |
| Female sex | 0.28 | [0.09–0.93] | 0.033 |
| Sepsis at admission (yes) | 1.09 | [0.17–7.2] | 0.93 |

e-Table 5. Univariate Poisson Regression Analysis for Dysphonia

| Variable | OR | 95% CI | p-value |
| --- | --- | --- | --- |
| Days under mechanical ventilation | 1.09 | [1.02–1.15] | 0.007 |
| Geniohyoid muscle stiffness (kPa) | 1.05 | [0.99–1.11] | 0.13 |
| Sternothyroid muscle stiffness (kPa) | 1.11 | [1.04–1.18] | 0.003 |
| Female sex | 3.23 | [1.24–8.37] | 0.016 |

e-Table 6. Multivariate Logistic Regression Analysis for Dysphonia.

# Supplemental Figures


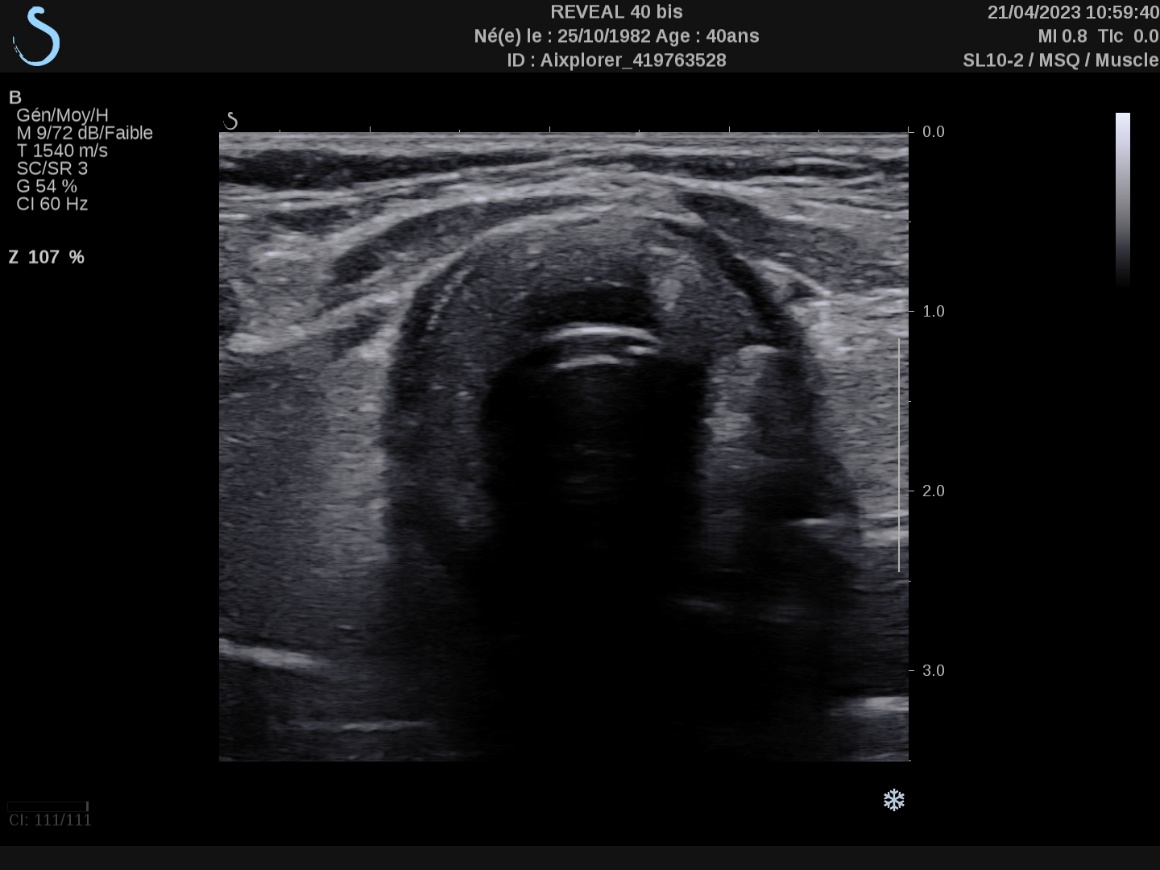

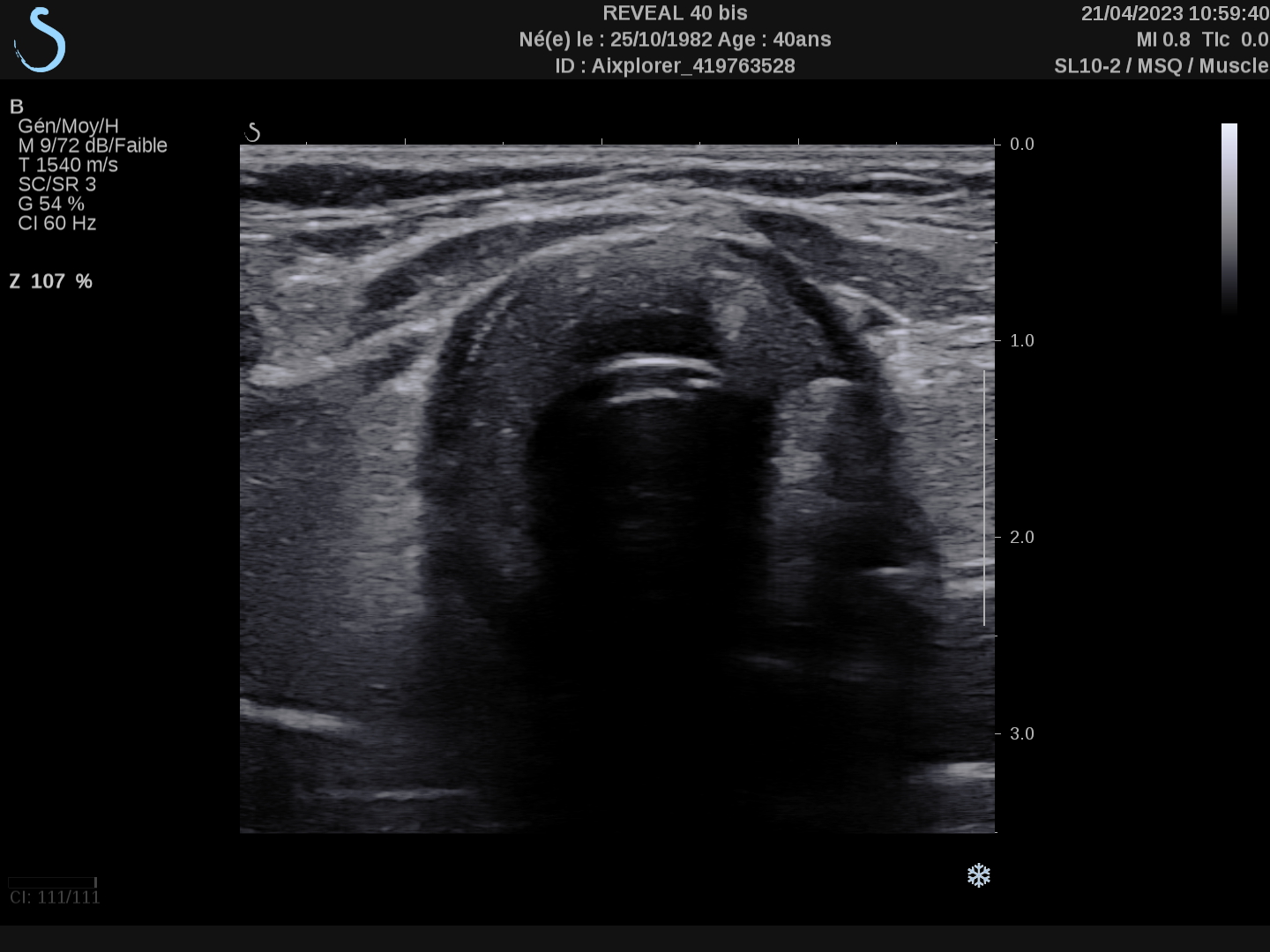


A

B

*

*

*

*

*

*

2

1

**e-Figure1.** **A**: Transverse view of the cricoid cartilage, obtained with the probe placed on the anterior neck at the level of cricoid cartilage. **B**: assessment of subglottic secretions (none, moderate, or high) in the area marked by the stars (*) around the intubation probe (1), and inside the cricoid cartilage (2).

# Appendix 1

|  | Author | Year | Population | Participants | Case | Percentage | 95%CI |
| --- | --- | --- | --- | --- | --- | --- | --- |
| Postextubation stridor | Colice et al. | 1989 | NS | 82 | 5 | 6.1% | 2-13.2% |
|  | Fisher et al. | 1992 | Mixed ICU | 62 | 7 | 11.3% | 3-8.3% |
|  | Marik et al. | 1996 | Mixed ICUs | 100 | 2 | 2% | 0.2-7% |
|  | Ho et al. | 1996 | NS | 38 | 10 | 26.3% | 13.4-43.1% |
|  | Miller and Cole | 1996 | Med. ICU | 100 | 6 | 6.0% | 2.2-12.6% |
|  | Epstein and Ciubotaru | 1998 | NS | 745 | N/A | N/A | N/A |
|  | Engoren et al. | 1999 | Cardio ICU | 531 | 3 | 0.6% | 0.1-1.6% |
|  | Sandhu et al. | 2000 | Trauma ICU | 110 | 13 | 11.8% | 6.4-19.3% |
|  | De Bast et al. | 2002 | Mixed ICU | 76 | 10 | 13.2% | 6.5-22.9% |
|  | Jaber et al. | 2003 | Mixed ICU | 112 | 13 | 11.6% | 6.3-19% |
|  | Maury et al. | 2004 | Med. ICU | 115 | 4 | 3.5% | 0.1-8.7% |
|  | Erginel et al. | 2005 | Resp. ICU | 67 | 7 | 10.4% | 4.3-20.3% |
|  | Kriner et al. | 2005 | Mixed ICUs | 462 | 20 | 4.3% | 2.6-6.6% |
|  | Ding et al. | 2006 | Med. ICU | 51 | 4 | 7.8% | 2.2-18.9% |
|  | Cheng et al. | 2006 | Mixed ICU | 236 | 18 | 7.6% | 4.6-11.8% |
|  | *Lim et al.* | *2006* | *Med. ICU* | *34* | *3* | *8.8%* | 1.9-23.7% |
|  | Lee et al. | 2007 | Med. ICU | 325 | 25 | 7.7% | 5-11.1% |
|  | Wang et al. | 2007 | Med. ICU | 110 | 20 | 18.2% | 11.5-26.7% |
|  | Shin et al. | 2008 | Trauma ICU | 49 | 1 | 2% | 0-10.9% |
|  | Sukhupanyarek et al. | 2008 | NS | 543 | 26 | 4.8% | 3.1-7% |
|  | Tadié et al. | 2010 | Med. ICU | 136 | 18 | 13.2% | 8-20.1% |
|  | Antonaglia et al. | 2010 | Mixed ICU | 42 | 2 | 4.8% | 0.6-16.2% |
|  | Cheng et al. | 2011 | NS | 113 | 16 | 14.2% | 8.3-22% |
|  | Gros et al. | 2012 | Mixed ICU | 104 | 7 | 6.7% | 2.7-13.4% |
|  | Keeratichananont et al. | 2012 | NS | 115 | 17 | 16.5% | 8.6-22.6% |
|  | Radhi et al. | 2012 | Med. ICU | 51 | 4 | 7.8% | 2.2-19.19% |
|  | Sutherasan et al. | 2013 | Mixed ICU | 101 | 16 | 15.8% | 9.3-24.5% |
|  | Mikaeili et al. | 2014 | NeuroMed | 41 | 4 | 9.8% | 2.7-23.1% |
|  | Abbasi et al. | 2014 | NS | 35 | 7 | 20% | 8.4-36.9% |
|  | Saeed et al. | 2014 | NS | 70 | 10 | 7% | - |
|  | Patel et al. | 2015 | Mixed ICUs | 51 | 2 | 3.9% | 0.5-13.5% |
|  | El-baradey et al. | 2016 | NS | 432 | 45 | 10.5% | 7.7-24.7% |
|  | Sahbal et al. | 2016 | NS | 50 | 4 | 8% | 2.2-19.2% |
|  | Schnell et al. | 2017 | NS | 362 | 34 | 9.4% | 6.6-12.9% |
|  | Samanta et al. | 2019 | NS | 52 | 11 | 21.2% | 11-34.7% |
|  | Tokunaga et al. | 2022 | NS | 25 | 0 | 0% | 0-13.7% |

**Appendix 1 references**

Abbasi S. Moradi S. Talakoub R. Kashefi P. Koushki AM. Effect of nebulized budesonide in preventing postextubation complications in critically patients: a prospective. randomized. double-blind. placebo-controlled study. Adv Biomed Res. 2014;3:182.

Antonaglia V. Vergolini A. Pascotto S. Bonini P. Renco M. Peratoner A. Buscema G. De Simoni L. Cuf-leak test predicts the severity of postextubation acute laryngeal lesions: a preliminary study. Eur J Anaesthesiol. 2010;27(6):534–41.

Cheng KC. Chen CM. Tan CK. Chen HM. Lu CL. Zhang H. Methylprednisolone reduces the rates of postextubation stridor and reintubation associated with attenuated cytokine responses in critically ill patients. Minerva Anestesiol. 2011;77:503–9.

Cheng KC. Hou CC. Huang HC. Lin SC. Zhang H. Intravenous injection of methylprednisolone reduces the incidence of postextubation stridor in intensive care unit patients. Crit Care Med. 2006;34:1345–50.

Chung YH. Chao TY. Chiu CT. Lin MC. The cuff-leak test is a simple tool to verify severe laryngeal edema in patients undergoing long-term mechanical

Colice GL. Stukel TA. Dain B. Laryngeal complications of prolonged intubation. Chest. 1989;96:877–84.

Darmon JY. Rauss A. Dreyfuss D. Bleichner G. Elkharrat D. Schlemmer B. et al. Evaluation of risk factors for laryngeal edema after tracheal extubation in adults and its prevention by dexamethasone. A placebo-controlled. double-blind. multicenter study. Anesthesiology. 1992;77:245–51.

De Bast Y. De Backer D. Moraine JJ. Lemaire M. Vandenborght C. Vincent JL. The cuff leak test to predict failure of tracheal extubation for laryngeal edema. Intensive Care Med. 2002;28:1267–72.

Ding LW. Wang HC. Wu HD. Chang CJ. Yang PC. Laryngeal ultrasound: a useful method in predicting post-extubation stridor. A pilot study. Eur Respir J. 2006;27:384–9.

El-Baradey GF. El-Shmaa NS. Elsharawy F. Ultrasound-guided laryngeal air column width diference and the cuf leak volume in predicting the efectiveness of steroid therapy on postextubation stridor in adult. Are they useful? J Crit Care. 2016;36:272–6.

Engoren M. Evaluation of the cuf-leak test in a cardiac surgery population. Chest. 1999;116(4):1029–31.

Epstein SK. Ciubotaru RL. Independent effects of etiology of failure and time to reintubation on outcome for patients failing extubation. Am J Respir Crit Care Med. 1998;158:489–93.

Erginel S. Ucgun I. Yildirim H. Metintas M. Parspour S. High body mass index and long duration of intubation increase post-extubation stridor in patients with mechanical ventilation. Tohoku J Exp Med. 2005;207(2):125–32.

François B. Bellissant E. Gissot V. Desachy A. Normand S. Boulain T. et al. 12-h pretreatment with methylprednisolone versus placebo for prevention of postextubation laryngeal oedema: a randomised double-blind trial. Lancet. 2007;369:1083–9.

Gros A. Holzapfel L. Marque S. Perard L. Demingeon G. Piralla B. et al. Intra-individual variation of the cuff-leak test as a predictor of post-extubation stridor. Respir Care. 2012;57:2026–31.

Ho LI. Harn HJ. Lien TC. Hu PY. Wang JH. Postextubation laryngeal edema in adults. Risk factor evaluation and prevention by hydrocortisone. Intensive Care Med. 1996;22:933–6.

Jaber S. Chanques G. Matecki S. Ramonatxo M. Vergne C. Souche B. et al. Post-extubation stridor in intensive care unit patients. Risk factors evaluation and importance of the cuff-leak test. Intensive Care Med. 2003;29:69–74.

Keeratichananont W. Limthong T. Keeratichananont S. Cuf leak volume as a clinical predictor for identifying post-extubation stridor. J Med Assoc Thailand. 2012;95(6):752–5.

Kriner EJ. Shafazand S. Colice GL. The endotracheal tube cuff-leak test as a predictor for postextubation stridor. Respir Care. 2005;50:1632–8.

Lee CH. Peng MJ. Wu CL. Dexamethasone to prevent postextubation airway obstruction in adults: a prospective. randomized. double-blind. placebo-controlled study. Crit Care. 2007;11:R72.

Lim SY. Suh GY. Kyung SY. An CH. Lee SP. Park JW. Jeong SH. Ham HS. Ahn YM. Lim SY. et al. Risk factors of extubation failure and analysis of cuf leak test as a predictor for postextubation stridor. Tuberc Respir Dis. 2006;61(1):34–40.

Maury E. Guglielminotti J. Alzieu M. Qureshi T. Guidet B. Offenstadt G. How to identify patients with no risk for postextubation stridor? J Crit Care. 2004;19:23–8.

Mikaeili H. Yazdchi M. Tarzamni MK. Ansarin K. Ghasemzadeh M. Laryngeal ultrasonography versus cuff leak test in predicting postextubation stridor. J Cardiovasc Thorac Res. 2014;6:25–8.

Miller RL. Cole RP. Association between reduced cuff leak volume and postextubation stridor. Chest. 1996;110:1035–40.

Patel AB. Ani C. Feeney C. Cuf leak test and laryngeal survey for predicting post-extubation stridor. Indian J Anaesth. 2015;59(2):96–102.

Radhi S. Guerra D. Alalawi R. Raj R. Nugent K. Cuf leak tests at the time of extubation correlate with voice quality assessment. ICU Dir. 2012;3(1):27–30

Sahbal MA. Mohamed KA. Zaghla HH. Kenawy MM. Laryngeal ultrasound versus cuf leak test in prediction of post-extubation stridor. Egypt J Crit Care Med. 2017;5(3):83–6.

Samanta S. Azim A. Baronia A. Poddar B. Gurjar M. Singh R: Clinical risk. cuf leak test and laryngeal ultrasound based scoring system to predict postextubation stridor in intensive care unit patients. Eur Respir J 2019. 54.

Sandhu RS. Pasquale MD. Miller K. Wasser TE. Measurement of endotracheal tube cuff leak to predict postextubation stridor and need for reintubation. J Am Coll Surg. 2000;190:682–7.

Schnell D. Planquette B. Berger A. Merceron S. Mayaux J. Strasbach L. Legriel S. Valade S. Darmon M. Meziani F. Cuf leak test for the diagnosis of post-extubation stridor. J intensive Care Med. 2017;34:391-6.

Shin SH. Heath K. Reed S. Collins J. Weireter LJ. Britt LD. The cuf leak test is not predictive of successful extubation. Am Surg. 2008;74(12):1182–5.

Sukhupanyarak S. Risk factors evaluation and the cuf leak test as predictors for postextubation stridor. J Med Assoc Thailand. 2008;91(5):648–53.

Sutherasan Y. Theerawit P. Hongphanut T. Kiatboonsri C. Kiatboonsri S. Predicting laryngeal edema in intubated patients by portable intensive care unit ultrasound. J Crit Care. 2013;28:675–80.

Tadié JM. Behm E. Lecuyer L. Benhmamed R. Hans S. Brasnu D. et al. Post-intubation laryngeal injuries and extubation failure: a fiberoptic endoscopic study. Intensive Care Med. 2010;36:991–8.

Wang CL. Tsai YH. Huang CC. Wu YK. Ye MZ. Chou HM. Shu SC. Lin MC. The role of the cuf leak test in predicting the efects of corticosteroid treatment on postextubation stridor. Chang Gung Med J. 2007;30(1):53–61.
